# Supplementary material for: Rice production threatened by coupled stresses of climate and soil arsenic
Source: Nat Commun. 2019 Nov 1;10:4985. doi: 10.1038/s41467-019-12946-4 (PMC6825132; doi:10.1038/s41467-019-12946-4)
Supplement: Supplementary file 1 — Supplementary Information [file 41467_2019_12946_MOESM1_ESM.docx]

**Rice production threatened by coupled stresses of climate and soil arsenic**

E. Marie Muehe^1^, Tianmei Wang^1^, Carolin F. Kerl^2^, Britta Planer-Friedrich^2^, Scott Fendorf^1^

^1^Earth System Science Department, Stanford University, Stanford, California 94305, USA

^2^Faculty for Biology, Chemistry and Earth Sciences, Bayreuth University, 95440 Bayreuth, Germany

**Supplementary Information**


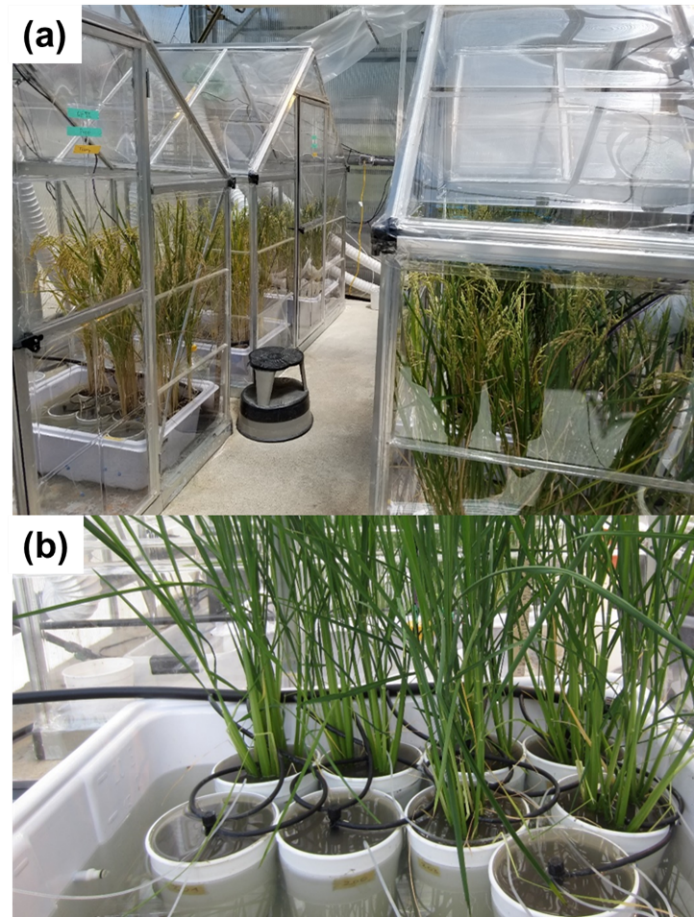


**Supplementary Figure 1 │ Custom-built growth chambers constructed in greenhouses for full climate control.** Four different climatic conditions were created in different growth chambers: (i) Today’s climate representing rice paddies at 33°C and 415 ppmv atmospheric CO_2_, (ii) future climatic conditions of 38°C and 850 ppmv CO_2_ based on RCP 8.5 projection of the International Panel on Climate Change (IPCC) report for the year 2100^1^, and (iii) elevated temperature or (iv) CO_2_ alone. In each growth chamber the medium Calrose grain *Oryza sativa* L. cv. M206 was grown on Californian paddy soil with either 7.3 or 24.5 mg As kg^-1^ dry soil (see Supplementary Table 1 for soil characterisation). Pots were placed in basins filled with water set to 5°C lower temperatures compared to the atmosphere, ensuring lower and constant temperatures in the soil compared to atmosphere. Water with a pH of 7 was used for irrigation and fed with an automatic irrigation system to prevent drying out of the pots. Rhizon samplers (used for collecting rhizosphere pore water) were placed at a depth of 10 cm in the pots.


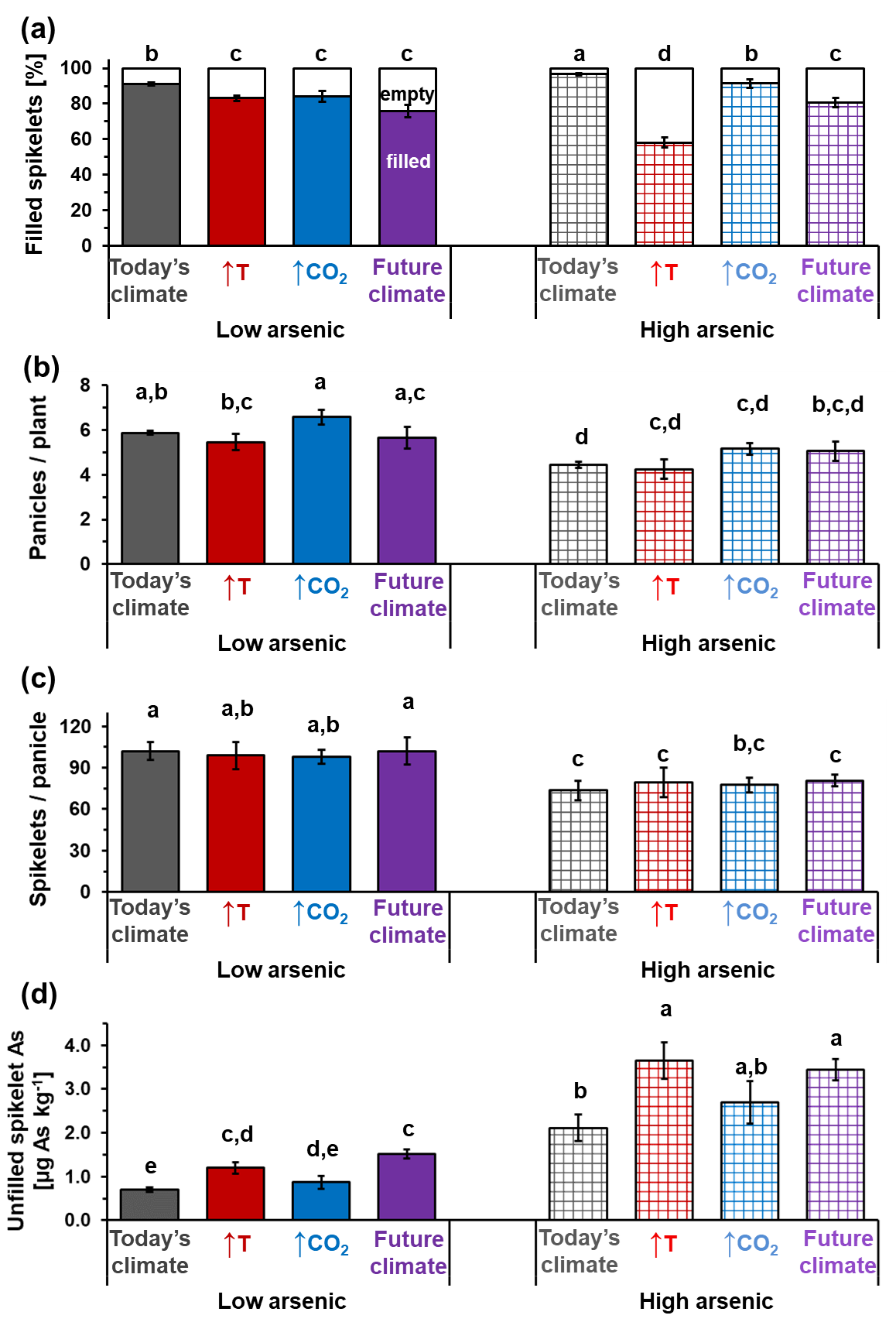


**Supplementary Figure 2 │** **Physiological traits determining grain yield in *Oryza sativa* L. cv M206 grown under different climatic and soil arsenic conditions.** (a) Percentage of spikelets filled (coloured) and unfilled (white), (b) number of panicles produced per plant, (c) number of spikelets produced per panicle, and (d) amount of arsenic accumulated in unfilled spikelets for rice grown under today’s (grey, 33°C and 415 ppmv CO_2_), future (purple, 38°C and 850 ppmv CO_2_), elevated temperature (red, 38°C and 415 ppmv CO_2_) and CO_2_ (blue, 33°C and 850 ppmv CO_2_) climatic conditions on paddy soil with low (solid, 7.3 mg As kg^-1^ dry soil) and high (checkered, 24.5 mg As kg^-1^ dry soil) arsenic levels. (8 biological replicates, mean values ± standard errors were compared to each other using the unpaired t-test at a 95% confidence interval. Different lowercase letters indicate that mean values were significantly different from each other (p < 0.05)).
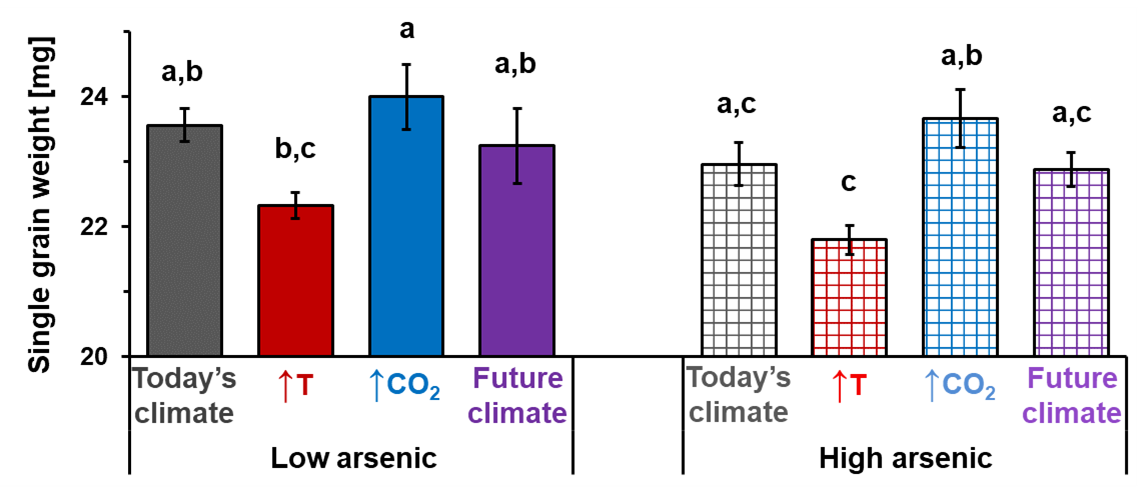


**Supplementary Figure 3 │** **Single grain weight of *Oryza sativa* L. cv M206 grown under different climatic and soil arsenic conditions.** Weight of single grains (mg) for rice grown under today’s (grey, 33°C and 415 ppmv CO_2_), future (purple, 38°C and 850 ppmv CO_2_), elevated temperature (red, 38°C and 415 ppmv CO_2_) and CO_2_ (blue, 33°C and 850 ppmv CO_2_) climatic conditions on paddy soil with low (solid, 7.3 mg As kg^-1^ dry soil) and high (checkered, 24.5 mg As kg^-1^ dry soil) arsenic levels. (8 biological replicates, mean values ± standard errors were compared to each other using the unpaired t-test at a 95% confidence interval. Different lowercase letters indicate that mean values were significantly different from each other (p < 0.05)).


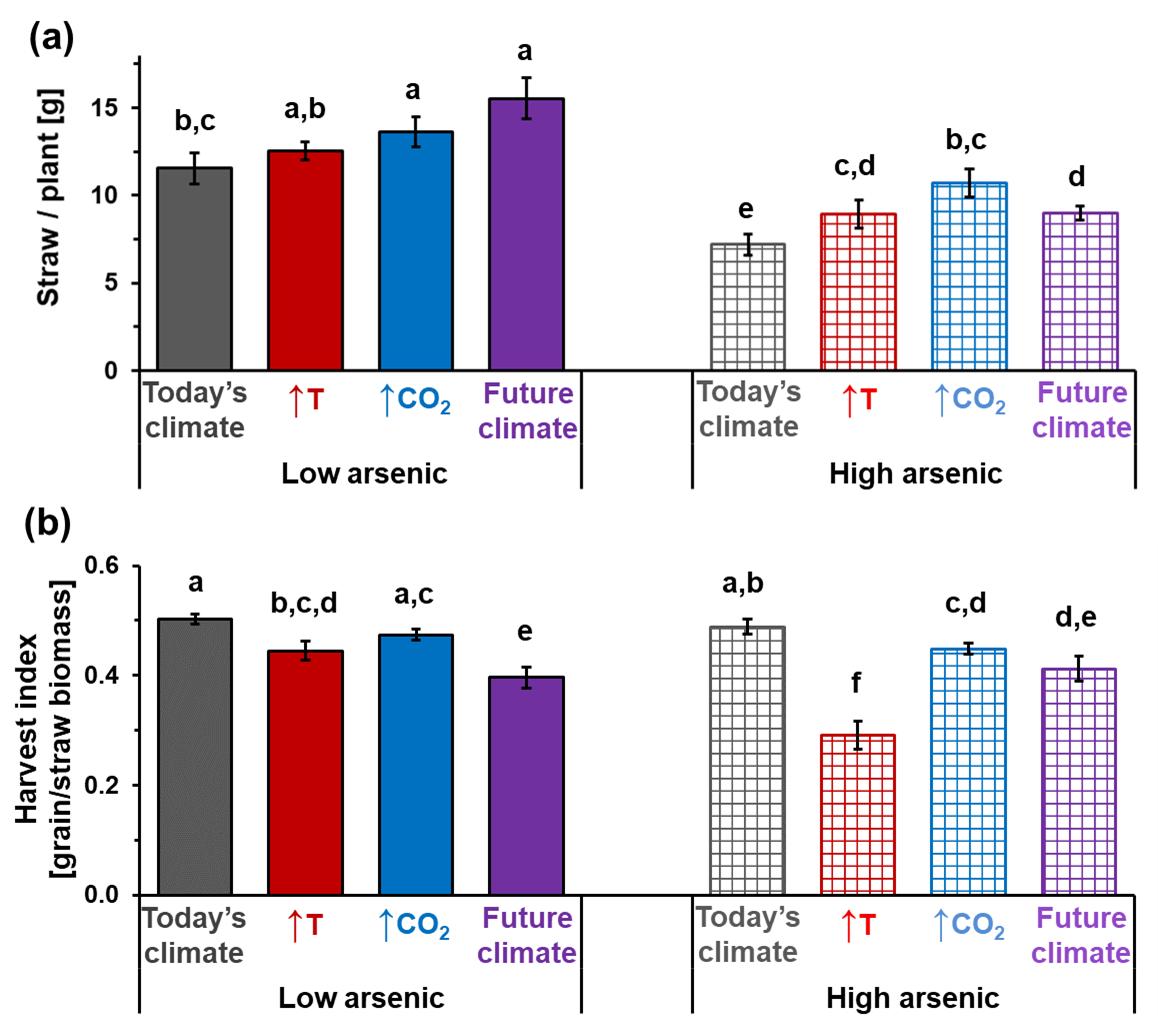


**Supplementary Figure 4 │** **Yield and** **Harvest index for *Oryza sativa* L. cv M206 grown under different climatic and soil arsenic conditions.** (a) Amount of straw biomass produced by rice and (b) calculated harvest index (amount of grain vs. amount of straw biomass) for rice grown under today’s (grey, 33°C and 415 ppmv CO_2_), future (purple, 38°C and 850 ppmv CO_2_), elevated temperature (red, 38°C and 415 ppmv CO_2_) and CO_2_ (blue, 33°C and 850 ppmv CO_2_) climatic conditions on paddy soil with low (solid, 7.3 mg As kg^-1^ dry soil) and high (checkered, 24.5 mg As kg^-1^ dry soil) arsenic levels. (8 biological replicates, mean values ± standard errors were compared to each other using the unpaired t-test at a 95% confidence interval. Different lowercase letters indicate that mean values were significantly different from each other (p < 0.05)).


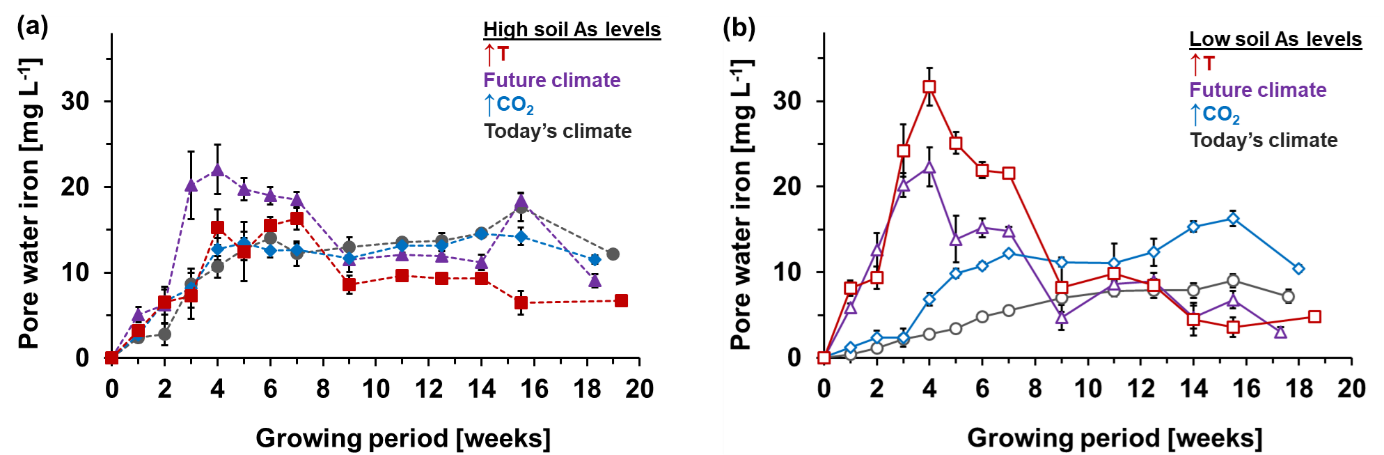


**Supplementary Figure 5 │ Dissolved iron concentrations in rhizosphere pore water of *Oryza sativa* L. cv. M206 under different climatic and soil arsenic conditions.** (a, b) Total pore-water iron. Pore water was extracted at 10 cm soil depth from M206 grown under today’s (grey), future (purple), elevated temperature (red) and elevated atmospheric CO_2_ (blue) climatic conditions on paddy soil with high (filled symbols with dashed line, 24.5 mg As kg^-1^ dry soil) and low (empty symbols with solid line, 7.3 mg As kg^-1^ dry soil) soil arsenic levels. (6 biological replicates, mean values ± standard errors).


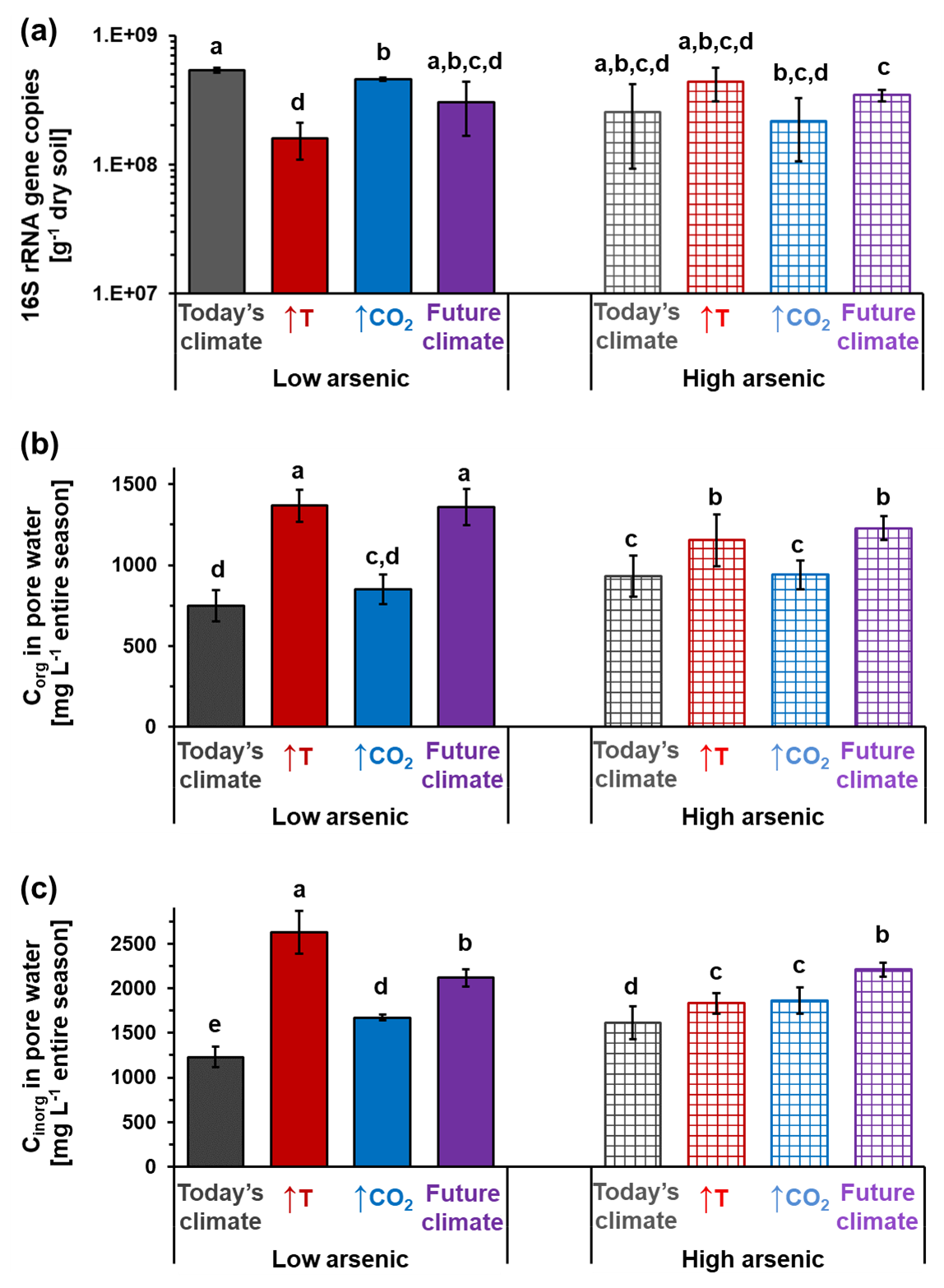


**Supplementary Figure 6 │ Microbial 16S rRNA gene copy numbers and respiration dynamics in *Oryza sativa* L. cv. M206 rhizospheres under different climatic and soil arsenic conditions.** (a) Bacterial 16S rRNA gene copy numbers in rhizosphere soil (3 biological replicates), (b) organic and (c) inorganic carbon in rhizosphere pore water. Rhizosphere soil and pore water were sampled at 10 cm soil depth from M206 grown under today’s (grey), future (purple), elevated temperature (red) and elevated atmospheric CO_2_ (blue) climatic conditions on paddy soil with high (checkered, 24.5 mg As kg^-1^ dry soil) and low (solid, 7.3 mg As kg^-1^ dry soil) soil arsenic levels (6 biological replicates). (Mean values ± standard errors were compared to each other using the unpaired t-test at a 95% confidence interval. Different lowercase letters indicate that mean values were significantly different from each other (p < 0.05)).

**
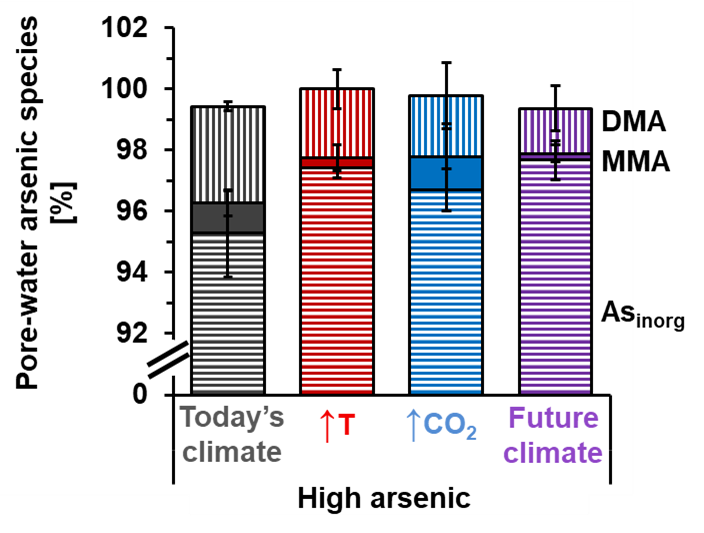
**

**Supplementary Figure 7 │ Dissolved arsenic species in rhizosphere pore water of *Oryza sativa* L. cv. M206 under different climatic and high soil arsenic.** Pore water was extracted at 10 cm soil depth from M206 grown under today’s (grey), future (purple), elevated temperature (red) and elevated atmospheric CO_2_ (blue) climatic conditions on paddy soil with high (24.5 mg As kg^-1^ dry soil) soil arsenic levels. (Inorganic arsenic = horizontally striped, MMA = solid fill, DMA = vertically stiped). (Mean values ± standard errors).

**Supplementary Table 1 │ Geochemical characterisation of Californian paddy soil.**

| 39°01'38.2"N 121°55'36.7"W, elevation: 22 m | | | |
| --- | --- | --- | --- |
| Property |  |  |  |
| Sand* | 31.5 | ±6.3 | % |
| Silt* | 20.17 | ±3.4 | % |
| Clay* | 48.4 | ±2.9 | % |
| Texture | clay | |  |
| pH [water]^†^ | 6.87 | ±0.03 |  |
| CEC^‡^ | 76.2 | ±11.7 | cMolc kg^-1^ dry soil |
| Total carbon^§^ | 1.34 | ±0.03 | % |
| Total nitrogen^§^ | 0.10 | ±0.01 | % |
| Si^‖^ | 251.13 | ±0.14 | mg g^-1^ dry soil |
| Al^‖^ | 84.49 | ±0.10 | mg g^-1^ dry soil |
| Ca^‖^ | 12.63 | ±0.01 | mg g^-1^ dry soil |
| K^‖^ | 10.95 | ±0.01 | mg g^-1^ dry soil |
| Mg^‖^ | 22.28 | ±0.10 | mg g^-1^ dry soil |
| Na^‖^ | 9.71 | ±0.15 | mg g^-1^ dry soil |
| Cl^‖^ | 45.65 | ±1.08 | µg g^-1^ dry soil |
| P^‖^ | 428.00 | ±4.29 | µg g^-1^ dry soil |
| Fe^‖^  Total  0.5 M HCl-extractable^¶^ | 54.44  4.99 | ±0.02  ±0.04 (71% Fe(II)) | mg g^-1^ dry soil  mg g^-1^ dry soil |
| Mn^‖^ | 789.88 | ±0.53 | µg g^-1^ dry soil |
| S^‖^ | 373.69 | ±2.50 | µg g^-1^ dry soil |
| As, native^‖^ | 7.26 | ±0.15 | µg g^-1^ dry soil |
| As, amended^‖^  Cd, native^‖^ | 24.55  2.91 | ±0.20  ±0.11 | µg g^-1^ dry soil  µg g^-1^ dry soil |

^*^ determined with hydrometer using 1N sodium hexametaphosphate

^†^ determined after 2, 24 and 48 h with deionized water at a 1:5 w/v ratio

^‡^ extracted for 4 h with 0.1 M BaCl_2_; pH 7, 1:25 w/v ratio, using Na, K, Ca, Mg, Al, NH_4_^+^

^§^ Determined by combustion (Carlo-Erba NA 1500, Danvers, MA, USA)

^‖^ Determined by X-ray fluorescence (Spectro XePositive HE XRF Spectrometer, AMETEK, Kleve, Germany)

^¶^ Extracted for 1 h with 0.5 M HCl, 1:40 w/v ratio, quantified with Ferrozine.

**Supplementary Table 2 │ Means and t-test p-values for yield and grain arsenic contents.** Means and standard errors are compared for (a) grain yield, (b) grain total arsenic content, (c) husk total arsenic content, and (d) arsenic retention by husk of rice calculated from eight biologically replicate plants grown under different climatic and soil arsenic conditions. Means were compared to each other using the unpaired t-test at a 95% confidence interval. The two-factorial ANOVA analysis for the interaction of soil As and climate is presented. Statistical significance is given as p-value and indicated with increasing depth of grey shading.

| (a) Grain yield (g plant^-1^) | | | | | | | | | | | | | | | | | | | | | | | | | | | | | |
| --- | --- | --- | --- | --- | --- | --- | --- | --- | --- | --- | --- | --- | --- | --- | --- | --- | --- | --- | --- | --- | --- | --- | --- | --- | --- | --- | --- | --- | --- |
|  |  | | | Low soil arsenic | | | | | | | | | | | | |  | High soil arsenic | | | | | | | | | | | |
|  | |  | | Today | | | ↑CO_2_ | | | | | ↑T | | | Future | |  | Today | | ↑CO_2_ | | ↑T | | | Future | | | | |
|  | g grain plant^-1^ | | | 11.46 | ±0.85 | | 12.43 | ±0.85 | | | | 9.96 | ±0.71 | | 9.64 | ±0.82 |  | 6.94 | ±0.55 | 8.72 | ±0.76 | 4.04 | ±0.48 | | 6.64 | | | ±0.36 | |
|  | %∆yield | | |  |  | | +8.5% | | | | | -13.1 | | | -15.9 | |  | -39.5 | | -23.9 | | -64.8 | | | -42.1 | | | | |
|  | |  | Low soil arsenic | | | | | | | | | | | | | |  | High soil arsenic | | | | | | | | | | | |
|  | | p-value |  | | | ↑CO_2_ | | | | ↑T | | | | Future | | |  | Today | | ↑CO_2_ | | ↑T | | | Future | | | | |
|  | | Low arsenic | Today | | | 0.2613 | | | | 0.1409 | | | | 0.0311 | | |  | 0.0000 | | 0.0055 | | 0.0000 | | | 0.0000 | | | | |
|  | |  | ↑CO_2_ | | |  | | | | 0.0113 | | | | 0.0026 | | |  | 0.0000 | | 0.0002 | | 0.0000 | | | 0.0000 | | | | |
|  | |  | ↑T | | |  | | | |  | | | | 0.3249 | | |  | 0.0003 | | 0.1406 | | 0.0000 | | | 0.0000 | | | | |
|  | |  | Future | | |  | | | |  | | | |  | | |  | 0.0394 | | 0.7962 | | 0.0000 | | | 0.0099 | | | | |
|  | | High arsenic | Today | | |  | | | |  | | | |  | | |  |  | | 0.0284 | | 0.0002 | | | 0.4729 | | | | |
|  | |  | ↑CO_2_ | | |  | | | |  | | | |  | | |  |  | |  | | 0.0000 | | | 0.0041 | | | | |
|  | |  | ↑T | | |  | | | |  | | | |  | | |  |  | |  | |  | | | 0.0009 | | | | |
|  | |  |  | | |  | | | |  | | | |  | | |  |  | |  | |  | | |  | | | | |
|  | | 2-factorial ANOVA | | | | | | | | | | | | | | |  |  | |  | |  | | |  | | | | |
|  | | Factor | df | | | Mean^2^ | | | | F-value | | | | p-value | | |  |  | |  | |  | | |  | | | | |
|  | | Soil As | 1 | | | 901.83 | | | | 94.43 | | | | 0.000 | | |  |  | |  | |  | | |  | | | | |
|  | | Climate | 3 | | | 115.92 | | | | 12.14 | | | | 0.000 | | |  |  | |  | |  | | |  | | | | |
|  | | Soil As x climate | 3 | | | 22.84 | | | | 2.39 | | | | 0.070 | | |  |  | |  | |  | | |  | | | | |
| (b) Grain total arsenic content (µg As kg^-1^ grain) | | | | | | | | | | | | | | | | | | | | | | | | | | | | | |
|  |  | | | Low soil arsenic | | | | | | | | | | | | |  | High soil arsenic | | | | | | | | | | | |
|  | |  | | Today | | | ↑CO_2_ | | | | ↑T | | | | Future | |  | Today | | ↑CO_2_ | | ↑T | | | | Future | | | |
|  | µg As kg^-1^ grain | | | 392.9 | ±11.9 | | 388.8± | 10.4 | | | 504.3 | | ±8.5 | | 579.7 | ±14.9 |  | 820.9 | ±21.5 | 987.3 | ±15.2 | 1038.8 | | ±23.3 | | 1003.9 | | | ±12.6 |
|  | |  | Low soil arsenic | | | | | | | | | | | | | |  | High soil arsenic | | | | | | | | | | | |
|  | | t-test, 95% CI |  | | | ↑CO_2_ | | | ↑T | | | | | Future | | |  | Today | | ↑CO_2_ | | ↑T | | | | Future | | | |
|  | | Low arsenic | Today | | | 0.7353 | | | 0.0000 | | | | | 0.0000 | | |  | 0.0000 | | 0.0000 | | 0.0000 | | | | 0.0000 | | | |
|  | |  | ↑CO_2_ | | |  | | | 0.0000 | | | | | 0.0000 | | |  | 0.0000 | | 0.0000 | | 0.0000 | | | | 0.0000 | | | |
|  | |  | ↑T | | |  | | |  | | | | | 0.0012 | | |  | 0.0000 | | 0.0000 | | 0.0000 | | | | 0.0000 | | | |
|  | |  | Future | | |  | | |  | | | | |  | | |  | 0.0000 | | 0.0000 | | 0.0000 | | | | 0.0000 | | | |
|  | | High arsenic | Today | | |  | | |  | | | | |  | | |  |  | | 0.0009 | | 0.0042 | | | | 0.0002 | | | |
|  | |  | ↑CO_2_ | | |  | | |  | | | | |  | | |  |  | |  | | 0.4814 | | | | 0.8454 | | | |
|  | |  | ↑T | | |  | | |  | | | | |  | | |  |  | |  | |  | | | | 0.5547 | | | |
|  | |  |  | | |  | | |  | | | | |  | | |  |  | |  | |  | | | |  | | | |
|  | | 2-factorial ANOVA | | | | | | | | | | | | | | |  |  | |  | |  | | | |  | | | |
|  | | Factor | df | | | Mean^2^ | | | F-value | | | | | p-value | | |  |  | |  | |  | | | |  | | | |
|  | | Soil As | 1 | | | 1.23x10^7 | | | 478.49 | | | | | 0.000 | | |  |  | |  | |  | | | |  | | | |
|  | | Climate | 3 | | | 3.64x10^5 | | | 14.10 | | | | | 0.000 | | |  |  | |  | |  | | | |  | | | |
|  | | Soil As x climate | 3 | | | 1.06x10^5 | | | 4.09 | | | | | 0.008 | | |  |  | |  | |  | | | |  | | | |
| (c) Husk total arsenic content (µg As kg^-1^ husk) | | | | | | | | | | | | | | | | | | | | | | | | | | | | | |
|  |  | | | Low soil arsenic | | | | | | | | | | | | |  | High soil arsenic | | | | | | | | | | | |
|  | |  | | Today | | | ↑CO_2_ | | | | ↑T | | | | Future | |  | Today | | ↑CO_2_ | | ↑T | | | | | Future | | |
|  | µg As kg^-1^ husk | | | 166.0 | ±49.7 | | 164.6 | ±96.1 | | | 472.0 | | ±74.2 | | 541.1 | ±113.4 |  | 1468.5 | ±214.5 | 1986 | .7±250.9 | 2023.9 | | ±229.1 | | | 1738.5 | | ±169.1 |
|  | |  | Low soil arsenic | | | | | | | | | | | | | |  | High soil arsenic | | | | | | | | | | | |
|  | | p-value |  | | | ↑CO_2_ | | | ↑T | | | | | Future | | |  | Today | | ↑CO_2_ | | ↑T | | | | | Future | | |
|  | | Low arsenic | Today | | | 0.9838 | | | 0.0138 | | | | | 0.0042 | | |  | 0.0003 | | 0.0000 | | 0.0001 | | | | | 0.0001 | | |
|  | |  | ↑CO_2_ | | |  | | | 0.0135 | | | | | 0.0048 | | |  | 0.0002 | | 0.0000 | | 0.0001 | | | | | 0.0000 | | |
|  | |  | ↑T | | |  | | |  | | | | | 0.8902 | | |  | 0.0016 | | 0.0000 | | 0.0003 | | | | | 0.0002 | | |
|  | |  | Future | | |  | | |  | | | | |  | | |  | 0.0018 | | 0.0000 | | 0.0004 | | | | | 0.0002 | | |
|  | | High arsenic | Today | | |  | | |  | | | | |  | | |  |  | | 0.0484 | | 0.1034 | | | | | 0.3424 | | |
|  | |  | ↑CO_2_ | | |  | | |  | | | | |  | | |  |  | |  | | 0.8965 | | | | | 0.2997 | | |
|  | |  | ↑T | | |  | | |  | | | | |  | | |  |  | |  | |  | | | | | 0.3783 | | |
|  | |  |  | | |  | | |  | | | | |  | | |  |  | |  | |  | | | | |  | | |
|  | | 2-factorial ANOVA | | | | | | | | | | | | | | |  |  | |  | |  | | | | |  | | |
|  | | Factor | df | | | Mean^2^ | | | F-value | | | | | p-value | | |  |  | |  | |  | | | | |  | | |
|  | | Soil As | 1 | | | 32.56 | | | 182.92 | | | | | 0.000 | | |  |  | |  | |  | | | | |  | | |
|  | | Climate | 3 | | | 0.56 | | | 3.12 | | | | | 0.033 | | |  |  | |  | |  | | | | |  | | |
|  | | Soil As x climate | 3 | | | 0.30 | | | 1.69 | | | | | 0.179 | | |  |  | |  | |  | | | | |  | | |

| (d) Arsenic retention by husk (%) ( Husk arsenic content / (Grain arsenic content + Husk arsenic content) *100) | | | | | | | | | | | | | | | | | | | | | | | | | | |
| --- | --- | --- | --- | --- | --- | --- | --- | --- | --- | --- | --- | --- | --- | --- | --- | --- | --- | --- | --- | --- | --- | --- | --- | --- | --- | --- |
|  |  | | | Low soil arsenic | | | | | | | | | | | | |  | High soil arsenic | | | | | | | | |
|  | |  | | Today | | | | ↑CO_2_ | | | ↑T | | | Future | | |  | Today | | ↑CO_2_ | | | ↑T | | Future | |
|  | % retention | | | 31.0 | ±5.0 | | | 32.1 | ±10.3 | | 47.9± | 8.5 | | 46.1± | | 8.8 |  | 62.9± | 4.3 | 66.7± | | 4.1 | 65.5± | 3.4 | 62.2± | 5.7 |
|  | |  | Low soil arsenic | | | | | | | | | | | | | |  | High soil arsenic | | | | | | | | |
|  | | p-value |  | | | | ↑CO_2_ | | | ↑T | | | Future | | | |  | Today | | ↑CO_2_ | | | ↑T | | Future | |
|  | | Low arsenic | Today | | | | 0.8754 | | | 0.0174 | | | 0.0135 | | | |  | 0.0000 | | 0.0000 | | | 0.0000 | | 0.0000 | |
|  | |  | ↑CO_2_ | | | |  | | | 0.0694 | | | 0.0888 | | | |  | 0.0024 | | 0.0014 | | | 0.0018 | | 0.0023 | |
|  | |  | ↑T | | | |  | | |  | | | 0.7891 | | | |  | 0.0268 | | 0.0104 | | | 0.0142 | | 0.0347 | |
|  | |  | Future | | | |  | | |  | | |  | | | |  | 0.0061 | | 0.0017 | | | 0.0026 | | 0.0094 | |
|  | | High arsenic | Today | | | |  | | |  | | |  | | | |  |  | | 0.2258 | | | 0.3714 | | 0.8480 | |
|  | |  | ↑CO_2_ | | | |  | | |  | | |  | | | |  |  | |  | | | 0.6596 | | 0.2278 | |
|  | |  | ↑T | | | |  | | |  | | |  | | | |  |  | |  | | |  | | 0.3510 | |
|  | |  |  | | | |  | | |  | | |  | | | |  |  | |  | | |  | |  | |
|  | | 2-factorial ANOVA | | | | | | | | | | | | | | |  |  | |  | | |  | |  | |
|  | | Factor | df | | | | Mean^2^ | | | F-value | | | p-value | | | |  |  | |  | | |  | |  | |
|  | | Soil As | 1 | | | | 9130.77 | | | 108.69 | | | 0.000 | | | |  |  | |  | | |  | |  | |
|  | | Climate | 3 | | | | 280.52 | | | 3.34 | | | 0.026 | | | |  |  | |  | | |  | |  | |
|  | | Soil As x climate | 3 | | | | 331.00 | | | 3.94 | | | 0.013 | | | |  |  | |  | | |  | |  | |
| p > 0.05 | | | | | | 0.05 ≥ p > 0.01 | | | | | | | | | 0.01 ≥ p > 0.001 | | | | | | p < 0.001 | | | | | |

**Supplementary Table 3 │ DMA and As(III)_i_ ratios in husk, bran and endosperm of *Oryza sativa* L. M206.** Ratios of DMA and As(III)_i_ (from X-ray fluorescence counts) in grains of rice produced under low and high soil arsenic and today’s and future climatic conditions (a) between and (b) within tissue type. X-ray fluorescent counts were obtained from images of grains (see Figure 3 in the main manuscript) taken at 12 500 eV at beamline 2-3 at the Stanford Synchrotron Radiation Lightsource (SSRL).

|  | Low arsenic | |  | High arsenic | |
| --- | --- | --- | --- | --- | --- |
|  | Today | Future |  | Today | Future |
| (a) Ratio between tissue type |  |  |  |  |  |
| DMA in husk/bran | 2.81 | 4.07 |  | 1.61 | 2.47 |
| DMA in bran/endosperm | 0.41 | 0.59 |  | 0.92 | 0.69 |
| As(III)_i_ in husk/bran | 0.49 | 0.42 |  | 0.40 | 0.38 |
| As(III)_i_ bran/endosperm | 1.35 | 1.47 |  | 1.75 | 2.18 |
| (b) Ratio within tissue type |  |  |  |  |  |
| As(III)_i_ / DMA in husk | 0.48 | 0.33 |  | 0.47 | 0.42 |
| As(III)_i_ / DMA in bran | 2.80 | 3.23 |  | 1.88 | 2.67 |
| As(III)_i_ / DMA in endosperm | 0.85 | 1.29 |  | 0.98 | 0.84 |

**Supplementary Table 4 │ Soil pore-water arsenic quantity and peak concentration.** The cumulative amount of arsenic released into the pore-water during the growing period and the week in which maximum arsenic concentrations were reached in the pore-water are provided (mean values ± standard errors).

|  | Total As released into pore water during season | |  | Maximum pore-water As level during season | | |
| --- | --- | --- | --- | --- | --- | --- |
|  | mg L^-1^ | |  | mg L^-1^ | | in week |
| Low soil arsenic |  |  |  |  | |  |
| Today's climate | 1.04 | ±0.07 |  | 0.17 | ±0.02 | 11 |
| eT | 1.61 | ±0.03 |  | 0.21 | ±0.01 | 4 |
| eCO2 | 1.19 | ±0.05 |  | 0.17 | ±0.00 | 9 |
| Future climate | 1.65 | ±0.07 |  | 0.20 | ±0.01 | 4 |
|  |  |  |  |  |  |  |
| High soil arsenic |  |  |  |  |  |  |
| Today's climate | 4.12 | ±0.29 |  | 0.52 | ±0.05 | 9 |
| eT | 6.98 | ±0.30 |  | 0.95 | ±0.05 | 6 |
| eCO2 | 5.13 | ±0.31 |  | 0.60 | ±0.02 | 9 |
| Future climate | 5.64 | ±0.19 |  | 0.69 | ±0.05 | 6 |

**Supplementary Discussion 1 │ Alternative approaches to greenhouse study**

Alternative approaches could have been field studies using either Open Top Chambers (OTC) or Free-Air Concentration Enrichment (FACE) set-ups. Both of these approaches, however, also have disadvantages and produce artefacts^2-5^. Similar to greenhouse studies, OTCs cause, for example, the acclimatization of rice to constantly high CO_2_, less water use as stomata close, loss in root growth. FACE studies usually focus on amending atmospheric CO_2_ up to only 600 sometimes 700 ppmv, which, according to current projections, will not be the likely scenario for the future^6^. They also shut off gas flow at night to save CO_2_, which creates artefacts. Most OTC and FACE studies, except Jasper Ridge, use CO_2_ as the only climate change proxy and do not incorporate temperature changes at the same time, which overestimates yields. Generally, studies looking at only one climate proxy do not give a full picture of climate change impacts on yields. In a greenhouse setting, we could fully control for atmospheric concentrations of CO_2_ and atmospheric and soil temperature, minimizing climate fluctuation impacts on arsenic movement from soil to plant.

**SUPPLEMENTARY REFERENCES:**

1 IPCC. Intergovernmental Panel on Climate Change: Climate Change 2013: The Physical Science Basis. (2013).

2 Baker, J. T. & Allen, L. H., Jr. in *CO_2_ and Biosphere* Vol. 14 *Advances in Vegetation Science* (eds J. Rozema, H. Lambers, S. C. Geijn, & M. L. Cambridge) Ch. 16, 239-260 (Springer Netherlands, 1993).

3 Long, S. P., Ainsworth, E. A., Leakey, A. D. B., Nösberger, J. & Ort, D. R. Food for thought: lower-than-expected crop yield stimulation with rising CO_2_ concentrations. *Science* **312**, 1918-1921 (2006).

4 Shaw, M. R. *et al.* Grassland responses to global environmental changes suppressed by elevated CO_2_. *Science* **298**, 1987-1990 (2002).

5 Ainsworth, E. A. & Long, S. P. What have we learned from 15 years of free-air CO_2_ enrichment (FACE)? A meta-analytic review of the responses of photosynthesis, canopy properties and plant production to rising CO_2_. *New Phytologist* **165**, 351-372 (2005).

6 Sanford, T., Frumhoff, P. C., Luers, A. & Gulledge, J. The climate policy narrative for a dangerously warming world. *Nature Climate Change* **4**, 164 (2014).
